# Fluorometric Detection of Oil Traces in a Sea Water Column

**DOI:** 10.3390/s22052039

**Published:** 2022-03-05

**Authors:** Emilia Baszanowska, Zbigniew Otremba

**Affiliations:** Department of Physics, Gdynia Maritime University, Morska 81-87, 81-225 Gdynia, Poland; z.otremba@wm.umg.edu.pl

**Keywords:** oil pollution in seawater, oil fluorescence, oil detection, seawater fluorescence, excitation–emission spectra, fluorometric index

## Abstract

This study focuses on broadening the knowledge of a fluorometric index to improve the detection of oil substances present in the marine environment. It is assumed that the value of this index will provide information about a possible oil discharge at some distance from the sensor. In this paper, the detection of oil present in seawater as a mixture of oils such as fuel, lubricate oil, or crude oil based on a fluorescence indicator-fluorometric index (FI_o/w_) is discussed. FI_o/w_ was defined based on specific excitation and emission wavelengths coming from the obtained excitation–emission spectrum (EEM) of oil-free seawater and, in parallel, the same water but artificially polluted with oil. For this, measurements of a mixture of oils in seawater for an oil-to-water ratio in the range from 50 × 10^−9^ to 200 × 10^−9^ as well as oil-free seawater were performed. Laboratory measurements continued five times in months in the summer season with the coastal waters of the southern Baltic Sea (last spring, summer, and early autumn). The dependence of FI_o/w_ on the presence of oil in seawater, the oil-in-water ratio, as well as months of the considered season has been demonstrated.

## 1. Introduction

The natural marine environment is still under threat of pollution resulting from human activities. Pollution is understood as elements, chemical compounds, and radionuclides that can already exist naturally in the marine environment but are introduced into it in excessive, harmful amounts. Chemical compounds and products completely unknown in this environment are also released into the sea (e.g., plastics) [1,2]. Pollutants are also different kinds of energies (e.g., acoustic, magnetic, electric, electromagnetic) [3]. Taking into account the impact of pollution on marine ecosystem health, it is necessary to strive for sustainable development. Therefore, various activities must be introduced to mitigate marine pollution.

Oil pollution can enter the seawater from oil spills, marine transport, run-offs or dumping, as well as from natural seepage from the bottom. Oil discharges consist of only 12% of oils entering marine waters. Generally, oil present in the seawater comes from traditional shipping, sewage systems, offshore drilling and oil extraction, as well as illegal discharges of various oil substances [4,5]. To reduce the amount of oil pollution, regulations were introduced to the MARPOL convention in the context of the construction of tanker ships, which are used to transport liquid cargoes, including crude oil, to standardize the methods of combatting oil spills at sea [6]. Despite the introduction of many regulations, offshore oil spills do occur, albeit in smaller quantities, and still pose a serious problem for the sea. However, a significant increase in routine shipping has recently been observed. This leads to an increased risk of an oil spill [7]. Therefore, it is necessary to detect and neutralize oil as soon as possible so that it does not penetrate deeper and to a large area.

Oils mainly consist of complex mixtures of monocyclic and polycyclic hydrocarbon compounds (aromatics). Aromatic compounds have the ability to light fluoresce [8]. In the authors’ studies, the fluorescence properties of oils are analyzed in relation to the possibilities of detecting oil substances in the water column.

When an oil spill appears on the surface of seawater as an oil plum, it becomes visible to the naked eye. In such cases, remote sensing technology allows spatially and near real-time measurements to detect and track oil pollutants. It was demonstrated that airborne and space-born remote detection is an effective tool to detect and map oil spills and to track pollutants [9,10,11,12,13,14,15,16]. The advantage of remote sensing is that it can track oil spills through space and time by using current information about the oil movement, rate, and direction. However, despite its many advantages, remote sensing has limitations; an example of this is the detection and identification of oil pollutants over the vertical dimension of the water column, when oil is not visible on the sea surface. Considering future remote technologies, this issue is developed by modeling the radiance reflectance of the water masses [17,18,19,20,21].

A relatively sensitive method to detect the oil present in the water column is fluorescence spectroscopy. However, seawater contains natural constituents such as dissolved organic matter (DOM) [22], colored dissolved organic matter (CDOM) [23,24,25,26], fluorescent dissolved organic matter (FDOM) [27,28], phytoplankton [29], and pigments [30], which absorb and fluoresce in the same spectral region as oil. This means that the fluorescence spectra of oils and natural seawater components partially overlap. The problem was studied by Baszanowska and Otremba in previous papers [31,32,33,34]. In these studies, it was indicated that oil fluorescence in the ultraviolet region and the fluorescence spectrum of natural seawater is disturbed. Moreover, the presence and content of natural constituents of seawater change depending on the season, temperature, and amount of light. Therefore, the fluorescence and absorption properties of natural seawater can also change due to constituents that can disturb the light attenuation in the water. One of the major constituents that can affect the light penetration and spectral quality of light attenuation in the water column is CDOM [35,36,37,38]. This is a major problem in detecting oil in seawater. 

In some situations when oil leaks from the pipeline, or it is released from wrecks or natural oil spills, oil is not visible to the naked eye. Moreover, such a situation is possible in accidental leaks or discharges of ballast water from ships, when a mixture of oil enters the water and penetrates to various depths and is present in the water column. In this case, oil can be present in seawater in two forms: as dissolved oil and as dispersed oil (oil-in-water emulsion). The measurements described in the paper laboratory are focused on the dissolved oil in seawater. 

In the authors’ previous studies, the excitation–emission spectra (EEMs) of several kinds of oils [33] were studied for an oil-to-water ratio ranging from 0.5 × 10^−6^ to 500 × 10^−6^. At that time, several types of crude oil, lubricating oil, and light and heavy fuel were tested. All of these types of oil produce a trace of their presence in the water, manifested by an increased intensity of fluorescence in the vicinity of the oil contamination. In the present study, various mixtures of oils were analyzed. 

The main aim of the research was to check the effectiveness of the fluorometric index as an indicator of oil detection in seawater potentially used in the future underwater sensor in the amount of oil estimated for an oil-to-water ratio ranging from 50 × 10^−9^ to 200 × 10^−9^ in seawater. Therefore, measurements of EEMs for oil-free seawater and seawater polluted with oil were performed in five different months for different oil-to-water ratios in the range from 50 × 10^−9^ to 200 × 10^−9^. 

## 2. Materials and Methods

### 2.1. Seawater Samples

**Figure 1:**
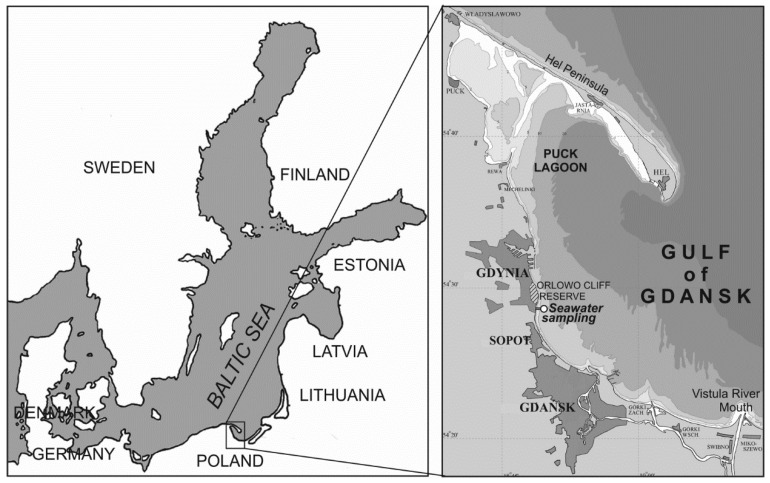
The area of the Baltic Sea where the seawater samples were taken.

Seawater was sampled from the Gdynia-Orłowo pier, located near the Gdynia Orłowo-Clif conservation area (Figure 1). Seawater was taken from a 1 m depth into 1-L glass bottles five times in 2019 in the period from May to October. These months were considered because they are in the summer season in the Baltic Sea basin. In this season, changes in the content of natural seawater constituents such as phytoplankton, CDOM, and other properties of seawater are observed. The properties of seawater for particular months in the time period from May to October are shown in Table 1.

### 2.2. Oil Samples and Preparing the Oil-Polluted Seawater Samples

For laboratory measurements, a mixture of oils consisting of seven different kinds of oils (crude oils, lubricate oils, and fuels) was used. The properties of the oils used to prepare the mixture of oil are described in Table 2.

Lubricate oils and fuels are used to prepare oil mixtures for use in ship engines. Oil Marinol 1240 is used to lubricate inverter-type marine engines, while lubricating oil Cyliten N460 is used in marine ship engine systems and for the lubrication of single- and multi-cylinder engines. E95 (gasoline) is used as a fuel for spark-ignition engines and Eurodiesel is used as fuel for diesel engines.

**Figure 2:**
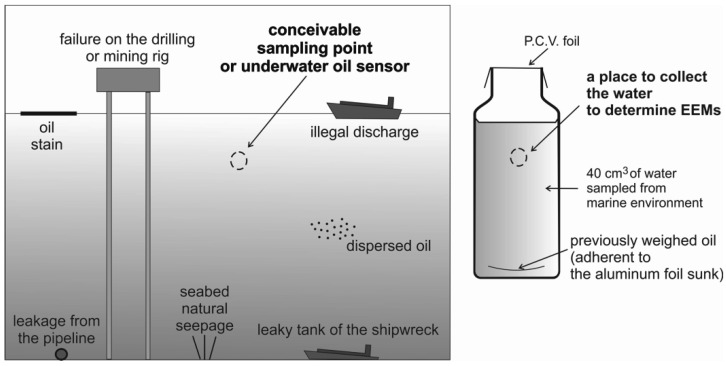
The possible situations of oil detection in the sea environment and principle of in-laboratory model realization.

For laboratory measurements, seawater samples for five different months in the summer season were contaminated by an oil mixture. The mixture of oils was prepared in such a way that each of the seven oils contributed approximately equally to the total volume of the oil mixture. From the prepared mixture of oils, an appropriate amount of oil was placed on a slice of aluminum foil and then weighed and inserted into a seawater sample to reach the desired oil-to-water ratio (r_o/w_). Samples polluted by a mixture of oil with r_o/w_ in the range of 50 × 10^−9^ to 200 × 10^−9^ were prepared. Natural seawater (oil-free seawater) for five different months in the summer season was exposed to the added oil for one day. The possible theoretical situation of oil detection in a marine environment and in laboratory realization of such contaminated seawater samples is presented in Figure 2. 

### 2.3. Measurement and Apparatus

A Hitachi F-7000 FL spectrofluorometer was used to determine the EEMs using a 1 × 1 cm quartz cuvette. 

The following measurement parameters were applied: the excitation wavelength was changed from 200 nm to 480 nm with an excitation sampling interval of 5 nm, the emission wavelength was changed from 260 nm to 600 nm with an emission sampling interval of 5 nm, the slit for excitation wavelength was set to 10 nm, the slit for emission wavelength was set to 10 nm, the integration time was set to 0.5 s, and the photomultiplier tube voltage was set to 400 V. 

The measurements were performed in several steps. Firstly, the temperature of the fluorimeter was stabilized at about 20 °C. Next, the EEMs for seawater samples were measured three times, allowing for a reliable, artifact-free background to be obtained. Finally, the EEMs of seawater contaminated by a mixture of oil for various r_o/w_ were measured. 

Rayleigh scattering to yield a digital matrix of EEMs was removed (if the excitation wavelength and emission wavelengths were equal and the emission wavelength was two times higher than the excitation wavelength).

## 3. Results and Discussion

### 3.1. EEM Spectra of Oil-Free Seawater and Polluted with Oil Seawater Samples 

**Figure 3:**
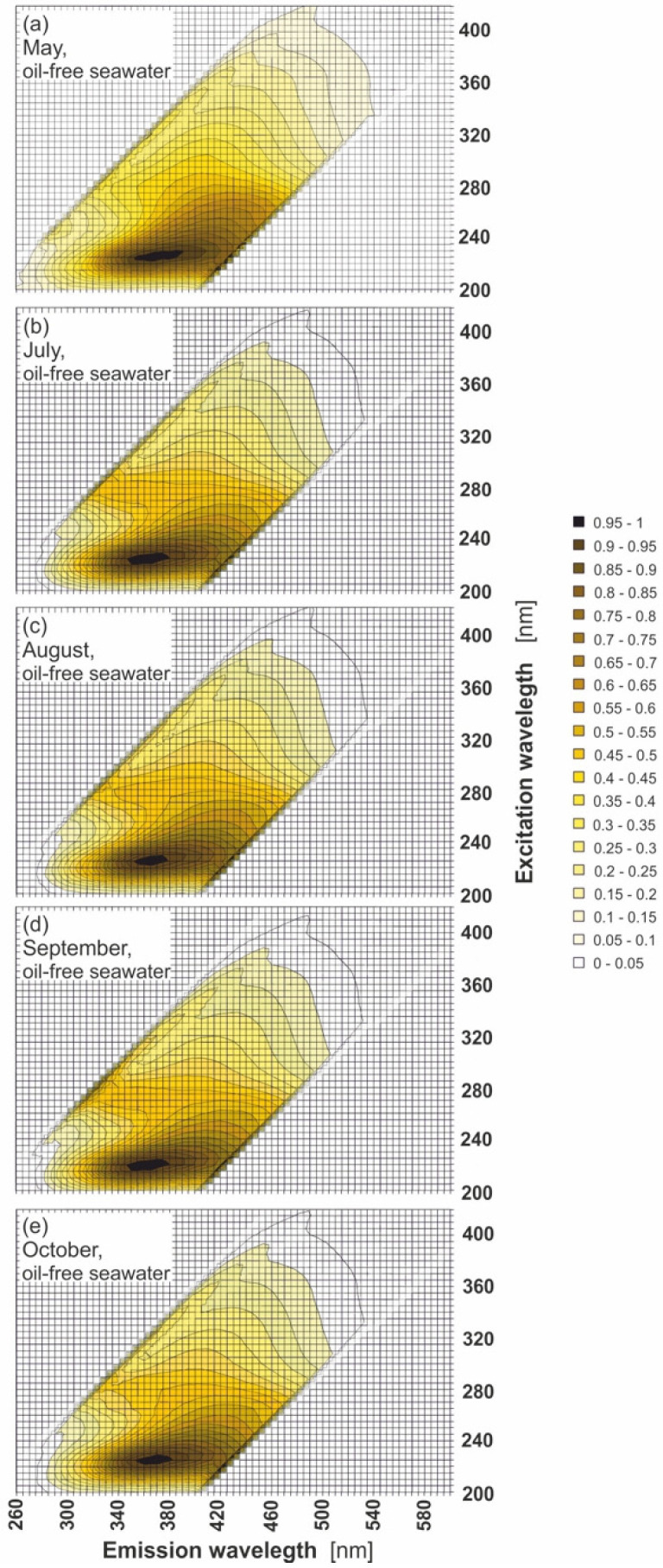
Excitation–emission spectra of oil-free seawater for various months: May (**a**), July (**b**), August (**c**), September (**d**), and October (**e**) in 2019.

Natural seawater components present in seawater ensure that natural seawater exhibits its own EEM spectra. Figure 3a–e presents EEMs for natural (oil-free) seawater samples in the period from May to October. The EEM spectra of oil-free seawater in May in Figure 3a indicate the presence of the main peak in the UV-range positioned at an excitation wavelength from 200 nm to 280 nm, centred at 225 nm, and corresponding to an emission wavelength from 300 nm to 420 nm, cantered at 365 nm. The entire spectral region is made up of peaks from various substances naturally found in seawater [22,23,25,31,40]. The shape of the EEM spectrum is created as a result of the combination of optical effects resulting from the fluorescence and absorption phenomena occurring in the spectrofluorometer cuvette. For July and August (Figure 3b,c), the position of the main peak is shifted towards lower wavelengths in the range of 5–10 nm, while for September and October, the position of the main peak is similar to May. The changes in the peaks in EEMs of natural seawater for different months depend on the changes of CDOM, the amount of which is variable [35] and determined by the optical properties of seawater in the Baltic Sea, especially in the coastal waters [26,41]. The changes in the CDOM peaks in EEMs correspond well to the changes in seasonal primary production (Table 1), which fluctuate and reach high values in May and October. However, their content is the lowest in July and is similar in August.

**Figure 4:**
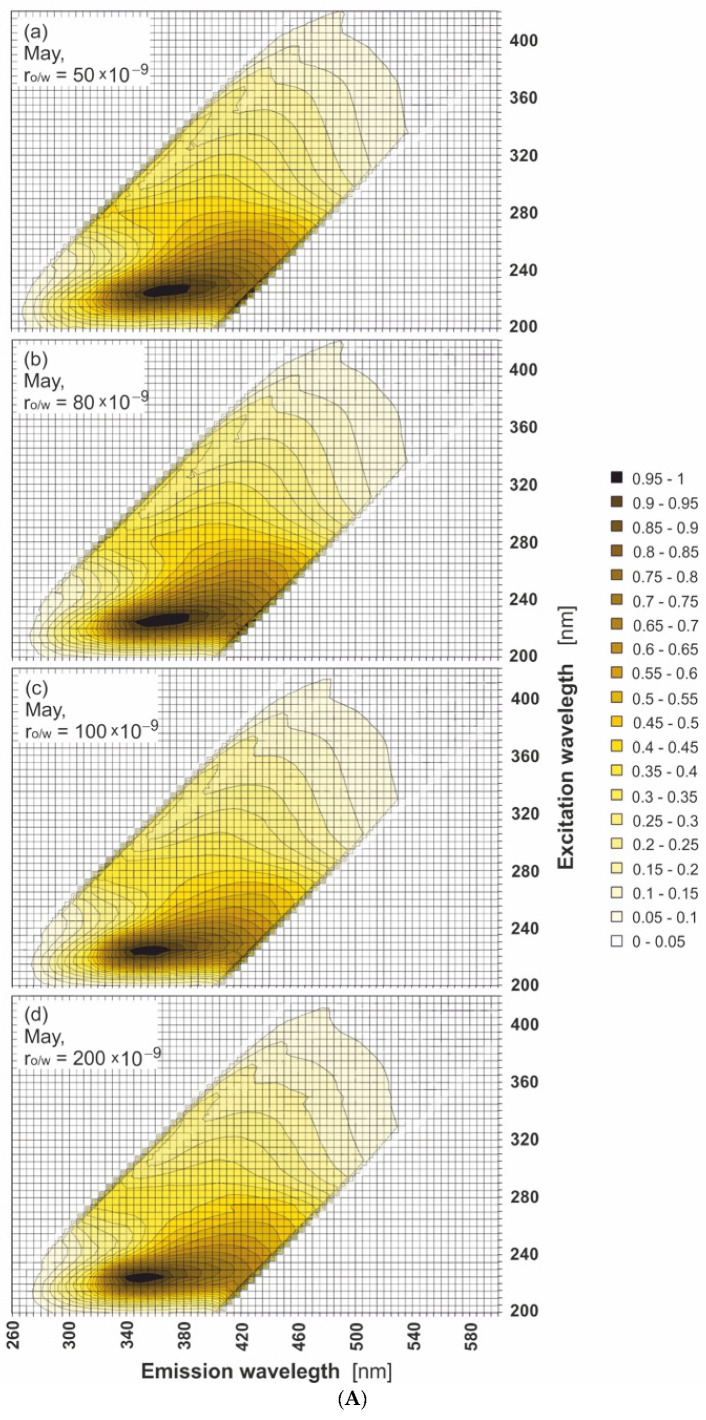

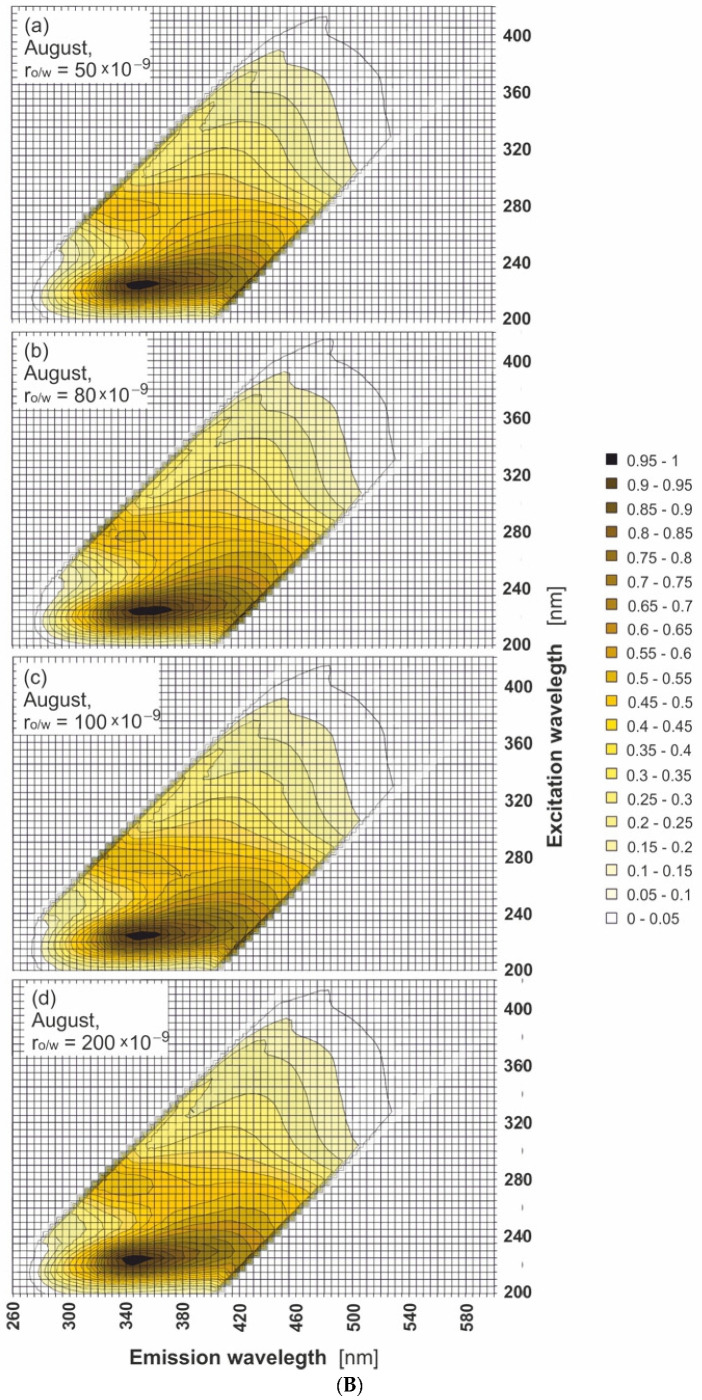

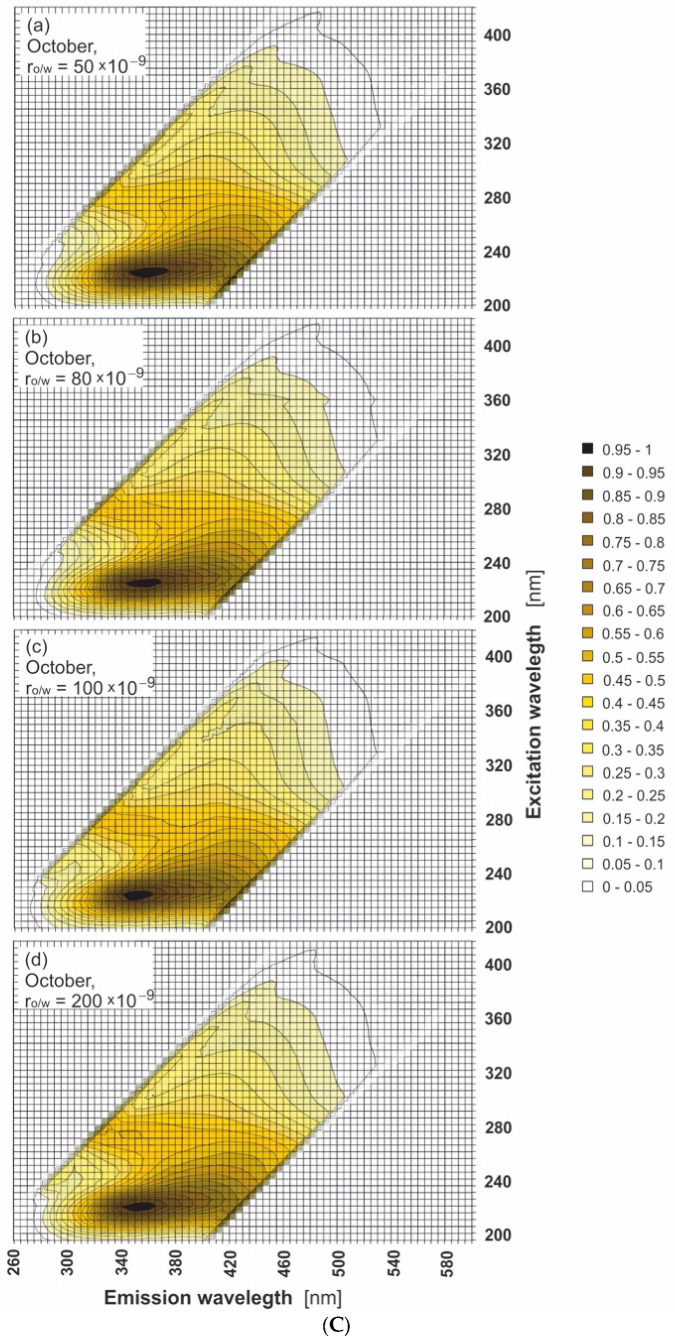
Excitation–emission spectra of seawater polluted with oil for various oil-to-water ratios: 50 × 10^−9^ (**a**), 80 × 10^−9^ (**b**), 100 × 10^−9^ (**c**), and 200 × 10^−9^ (**d**), for selected months: May (**A**), August (**B**), and October (**C**).

**Figure 5:**
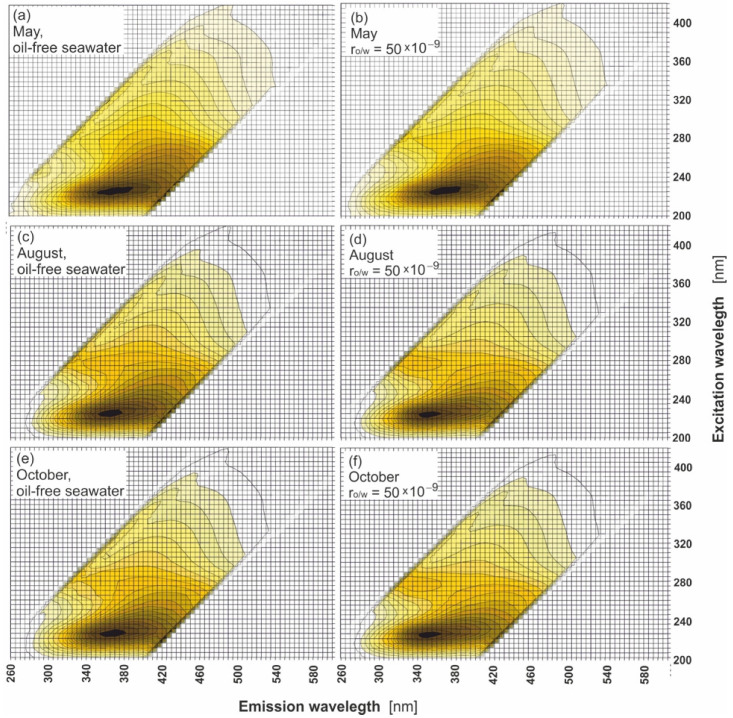
Excitation–emission spectra of oil-free seawater (**a**,**c**,**e**) and polluted seawater (**b**,**d**,**f**) for various months.

Figure 4 presents EEM spectra of seawater samples polluted by the mixture of oils for several r_o/w_: 50 × 10^−9^, 80 × 10^−9^, 100 × 10^−9^, and 200 × 10^−9^ in May (A), August (B), and October (C). In these figures, the shift in the main peak determined for oil-free seawater towards lower emission wavelengths for polluted seawater (r_o/w_ = 200 × 10^−9^) is visible. However, for lower r_o/w_ = 80 × 10^−9^ the changes in EEMs are hardly noticeable, while for the lowest r_o/w_ = 50 × 10^−9^ the EEM spectrum of seawater after contamination by a mixture of oil is the same as the EEM spectrum for oil-free seawater. This indicates that, for the considered range of r_o/w_, a high similarity of EEMs of seawater polluted by oil and oil-free seawater is observed. This is the confirmation that the detection of oil in that considered range of r_o/w_ based on EEMs is difficult, especially for r_o/w_ = 50 × 10^−9^. The same EEMs were determined for other months in the considered season between May and October. The EEMs of the lowest r_o/w_ = 50 × 10^−9^ was particularly considered in all months of sampling time to confirm the similarity between the EEMs of oil-polluted seawater and oil-free seawater. Figure 5a–f presents the EEMs of oil-free seawater and seawater polluted by the mixture of oils for r_o/w_ = 50 × 10^−9^ in May, August, and October. A comparison of the EEMs for seawater of the mixture of oils to the oil-free seawater indicates that peaks in EEMs of natural seawater components partially overlap with the peaks in EEMs for seawater polluted by the mixture of oils. 

**Figure 6:**
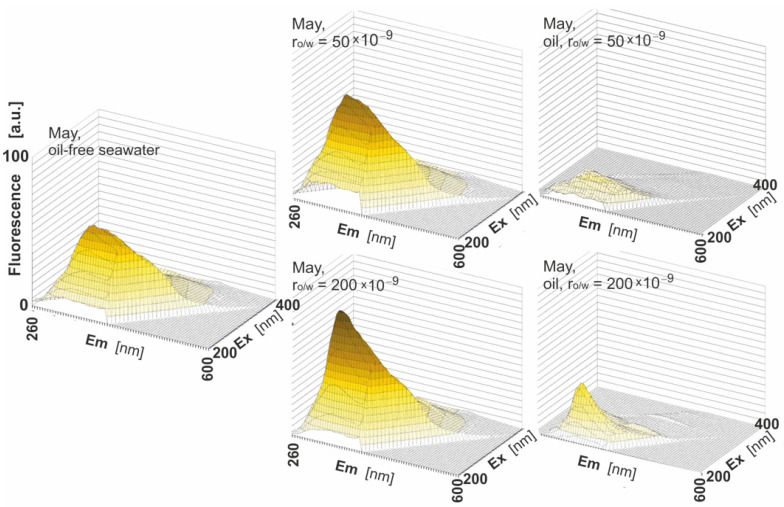
Excitation–emission spectra of oil-free seawater (**left**) compared with the excitation–emission of seawater contaminated with oil (charts in the **central column**), the same as in the case of the central column but after deducting the oil-free water fluorescent component (**right column**).

When EEMs are presented in 3D in Figure 6, the intensity of fluorescence is considered for two different r_o/w_, 50 × 10^−9^ and 200 × 10^−9^, for the example month. In this figure, the fluorescence intensity changes are observed for the highest r_o/w_ (200 × 10^−9^). To minimize the effect of seawater components on the EEM spectrum, the EEM spectrum of oil-free seawater was subtracted from the EEM spectrum of seawater polluted by oil to obtain the EEM spectrum of only the oil component (see the right side of Figure 6).

**Figure 7:**
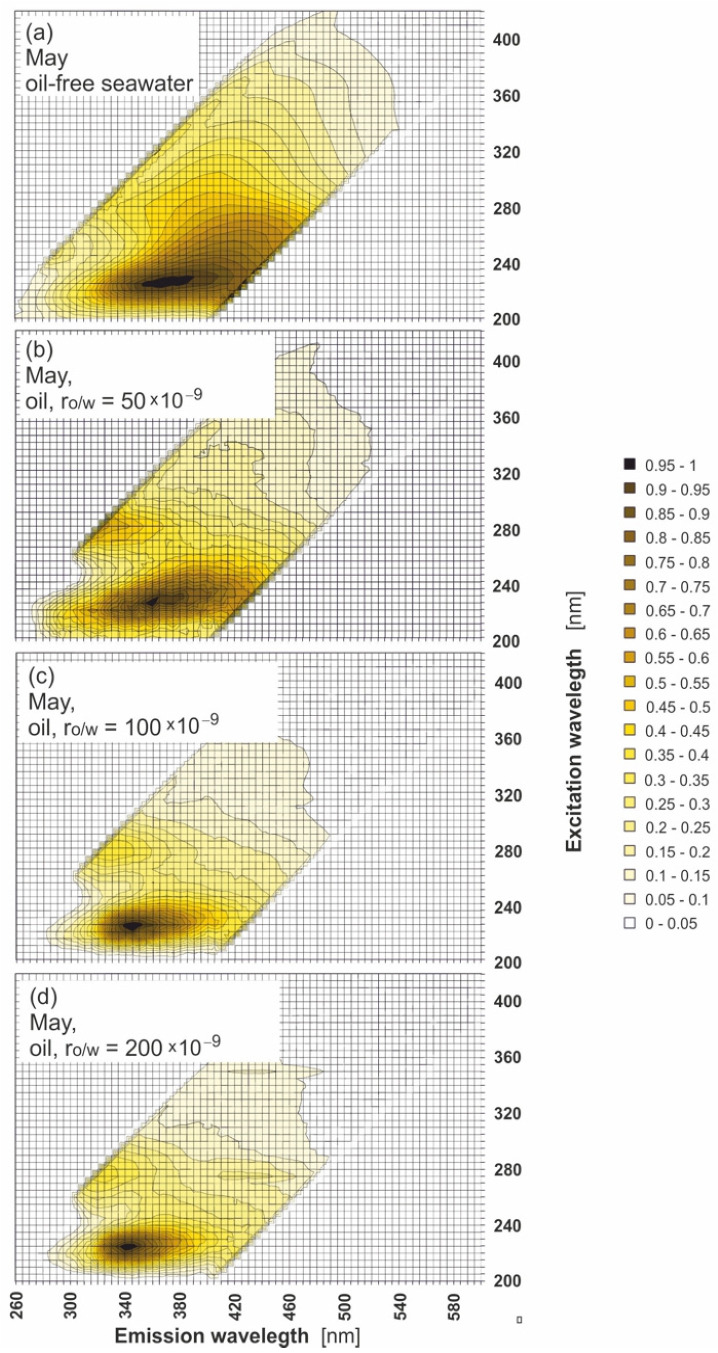
Excitation–emission spectra of oil-free seawater (**a**) in comparison with the excitation–emission of seawater polluted with oil but after deducting the oil-free water fluorescent component (**b**–**d**).

Figure 7 presents example EEMs of oil-free seawater (Figure 7a) shown in comparison with polluted examples (Figure 7b–d) but with the fluorescent component of seawater subtracted for various r_o/w_ for the example month (May). In this figure, the spectra shape dependence on r_o/w_ is observed. In the EEMs of oil, the main peak (centred at 340 nm for the emission wavelength) corresponds to the excitation wavelength at 225 nm for all r_o/w_ determined. The position of the main peak (λ_Ex_/λ_Em_ = 225/340) was also determined for each month in the sampling time, and a shift to a longer wavelength is observed when r_o/w_ decreases. The second peak centered at 275 nm for an excitation wavelength corresponding to an emission wavelength centred at 325 nm (λ_Ex_/λ_Em_ = 275/325) was also determined for each month in the sampling time. The third peak centred at 275 nm for excitation wavelength corresponding to an emission wavelength and centered at 420 nm (λ_Ex_/λ_Em_ = 275/425), disappearing when r_o/w_ decreases and is not noticeable for the lowest r_o/w_ (50 × 10^−9^). 

### 3.2. Calculation of the Fluorometric Index for Natural Seawater and Seawater Polluted with Oil

Oil detection based only on the shape of EEMs in the analyzed r_o/w_ range from 50 × 10^−9^ to 200 × 10^−9^ would be difficult due to the high similarity between EEMs of oil-free seawater and polluted seawater. The potential sensor would work based on fluorometric index FI_o/w_ calculated for a specific wavelength for both oil-free seawater and polluted seawater [33]. Formula (1) describes the FI_o/w_ definition as the quotient of the fluorescence intensity at the emission wavelength for seawater polluted by oil to the intensity at the emission wavelength for oil-free seawater corresponding to the detected excitation maxima for both waters.

(1) $${\mathrm{FI}}_{o/w}={\left[\frac{I(\lambda_{Emission\;\;of\;seawater\;polluted\;by\;oil})}{I(\lambda_{Emission\;\;of\;natural(oil-free)\;seawater})}\right]}_{\lambda_{Excitation}}$$

The specific excitation and emission wavelengths for FI_o/w_ definition were selected based on the EEMs of both oil-free seawater and polluted seawater. It was determined that the main peaks for oil-free seawater and polluted seawater were detected at the same excitation wavelength (225 nm), which corresponds to the emission wavelengths for the peaks detected for natural seawater at 355 nm and seawater polluted with oil at 340 nm, respectively.

Therefore, FI_o/w_ was calculated as the quotient of the fluorescence intensity at 340 nm emission wavelength and the intensity at 355 nm, while the excitation wavelength remained equal to 225 nm (Formula (2)).

(2) $${\mathrm{FI}}_{o/w}={\left[\frac{I\left(\lambda_{Em=340}\right)}{I\left(\lambda_{Em=355}\right)}\right]}_{\lambda_{Ex=225}}$$

where *I*(λ*_Em_*_=340_) describes the fluorescence intensity corresponding to the emission wavelength for polluted seawater (340 nm), and *I*(λ*_Em_*_=355_) describes the fluorescence intensity corresponding to the emission wavelength for oil-free seawater (355 nm) linked to the same excitation wavelength (λ*_Ex_*_=225_) (225 nm).

The data taken from EEM spectra of oil-free seawater and polluted seawater were used for calculation of the FI_o/w_. These calculations were performed based on formula (2) for all the considered months and various r_o/w_: 50 × 10^−9^, 80 × 10^−9^, 100 × 10^−9^, and 200 × 10^−9^. The results of FI_o/w_ calculations are presented in Table 3 for oil-free seawater for all considered months and in Table 4 for seawater polluted with oil for all considered r_o/w_. The obtained FI_o/w_ values for polluted seawater achieved higher values (in the range from 1.07 to 1.73) than for natural seawater (in the range from 0.83 to 0.87). The exception was the FI_o/w_ value determined for r_o/w_ = 50 × 10^−9^ in May when the FI_o/w_ value dropped below 1, but it was still higher than for oil-free seawater. 

**Figure 8:**
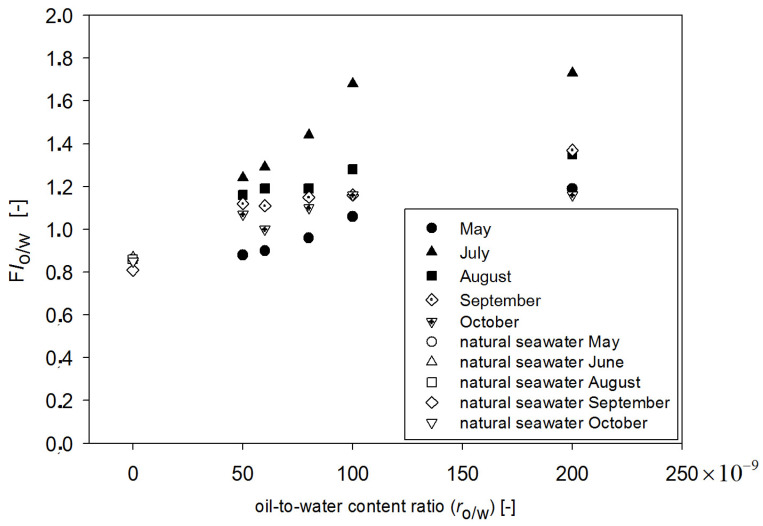
Fluorescence index for both oil-free seawater and seawater polluted with oil as a function of the oil-to-water ratio for May, July, August, September, and October.

**Figure 9:**
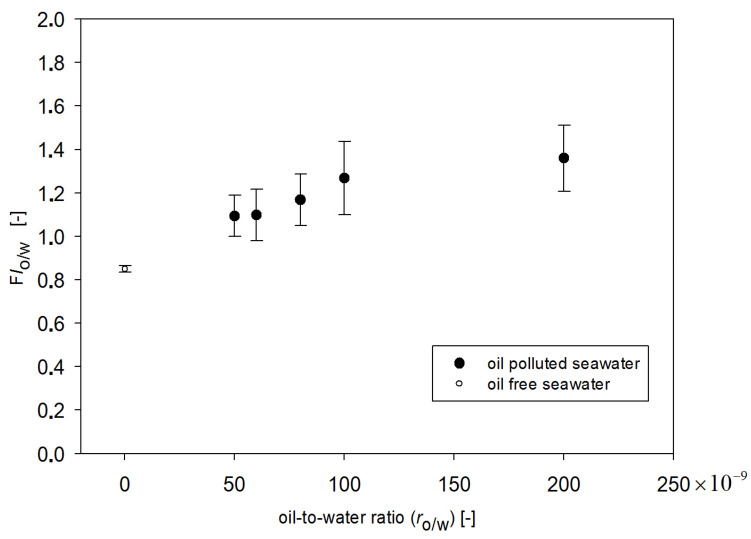
Fluorometric index FI with a standard deviation of the average for oil-free seawater and seawater artificially polluted by a mixture of oil.

This means that FI_o/w_ can be an indicator sensitive to the oil present in seawater. However, FI_o/w_ values depend on the r_o/w_ as well as on the date of sampling. In Figure 8, the dependence of FI_o/w_ values on the r_o/w_ and date of sampling are shown. The highest values of FI_o/w_ were obtained for July, while the lowest values were obtained for May data. Taking into account all considered months, the FI_o/w_ values for the lowest r_o/w_ = 50 × 10^−9^ are close to FI_o/w_ values of oil-free seawater in May, although oil is still detectable. When r_o/w_ for the range of 50 × 10^−9^ to 100 × 10^−9^ is considered, the proportional relation is observed between FI_o/w_ and r_o/w_. However, in the case when r_o/w_ for range from 100 × 10^−9^ to 200 × 10^−9^ is considered, FI_o/w_ is constant in relation to the r_o/w_. To check the deviation of determined fluorometric indexes (FI_o/w_) values, statistical calculations at different times of sampling for natural seawater and the same seawater polluted by oil were performed. The standard deviation of the average values of FI_o/w_ for a mixture of oils for 25 samples from five different times of seawater sampling from May to October were determined. Figure 9 presents the average standard deviation of the mean calculated FI_o/w_ values for natural seawater (oil-free seawater) and artificially polluted seawater by a mixture of oils for different r_o/w_. The highest value of the standard deviation of FI_o/w_ value was determined for the r_o/w_ = 100 × 10^−9^, and the standard deviation of the average FI_o/w_ value for oil-free seawater does not overlap with the standard deviation of the average FI_o/w_ value for oil-polluted seawater. Taking into account the standard deviations of FI_o__/w_, it can be concluded that the constant values of FI_o/w_ in the r_o/w_ are from 100 × 10^−9^ to 200 × 10^−9^.

However, taking into account the obtained results separately for each month (Figure 8), FI_o/w_ for May and October was lower than for other months. This suggests that the detection of oil is affected by the presence of natural seawater components. Taking into account the primary production (see Table 1), it achieved higher values in October and in May, and it corresponds with the low values of FI_o/w_ for those months. 

**Figure 10:**
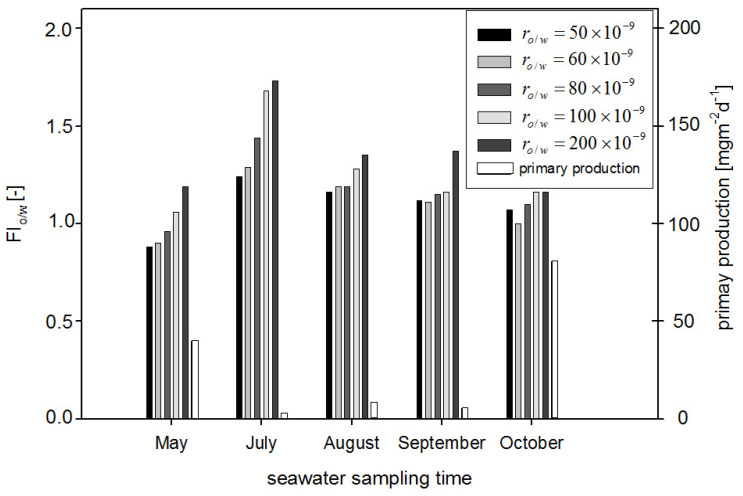
Fluorescence index of seawater polluted with oil in comparison with primary production.

The dependence of FI_o/w_ values on the primary production is presented in Figure 10, in which it is shown that FI_o/w_ achieved lower values when primary production achieved higher values in the particular months of May and October, respectively. However, in July, primary production achieved the lowest value (2.35), and it corresponds to the highest values of FI_o/w_. The results indicate that the primary production can probably disturb oil detection.

## 4. Conclusions

The main goal of the study was to analyze the effectiveness of the detection of traces of oil in the seawater based on fluorescence in relation to the low portion of oil in contact with seawater (r_o/w_ range from 50–200 × 10^−9^). To determine this, the EEM determinations of natural (oil-free) seawater and the same water with the addition of oil were carried out five times in the summer season months, using the coastal seawater from the southern Baltic Sea. The study results indicate that the possibility of detection of oil on the EEMs shape is practically impossible, especially for the lowest r_o/w_. However, the detection of oil becomes possible when FI_o/w_ is considered. It was shown that although the FI_o/w_ values increased when r_o/w_ increased, the FI_o/w_ values depended on the date of seawater sampling. It was also shown that the FI_o/w_ increased when natural seawater constituents were present in seawater at low levels. When the content of natural seawater constituents had a high level (May and October), FI_o/w_ achieved lower values, and the FI_o/w_ values for the lowest r_o/w_ were close to the FI_o/w_ values for the natural seawater. 

The results can provide data to plan an oil underwater sensor based on signaling a change in the fluorescent index when oil is present nearby.

## Figures and Tables

**Table 1:** Parameters of seawater sampled from Gdynia station, located in the coastal waters of the Gulf of Gdansk in the Southern Baltic Sea in Poland [39].

	May	July	August	September	October
Temperature T [°C]	8.44	20.4	17.8	17.4	14.3
Salinity [PSU]	6.1	6.17	5.85	6.13	6.02
Primary production [mg·m^−2^·d^−1^]	40	2.35	8.32	5.3	80.6

**Table 2:** Properties of several kinds of oils used to prepare the mixture of oils.

	Type of Oil	American Petroleum Institute (API) Gravity [°]	Extraction	Sulphur Content [%]	Polycyclic Aromatic Hydrocarbon (PCA) [%]
Crude oil					
Petrobaltic	Light crude	43–44	Baltic Sea	0.12	
Flotta	Medium crude	35.4	North Sea Orkney	1.22	
Gulfaks	Light crude	37.5	North Sea Offshore	0.22	
Lubricate oil					
Marinol 1240	Heavy		Commercial Lotos SA		<3
Cyliten N460	Heavy		Commercial Lotos SA		<3
Fuel					
E95	Light		Commercial Lotos SA		up to 1% benzene, <3% n-hexane, 6% toluene
Eurodiesel	Light		Commercial Lotos SA		<3

**Table 3:** FI_o/w_ for natural seawater sampled in May, July, August, September, and October 2019.

			FI_o/w_ [-]		
r_o/w_	May	July	August	September	October
natural seawater	0.86	0.87	0.86	0.81	0.85

**Table 4:** FI_o/w_ for seawater polluted by a mixture of oils for various oil-to-water-ratios r_o/w_ for May, July, August, September, and October 2019.

			FI_o/w_ [-]		
r_o/w_	May	July	August	September	October
200 × 10^−9^	1.19	1.73	1.35	1.37	1.16
100 × 10^−9^	1.06	1.68	1.28	1.16	1.16
80 × 10^−9^	0.96	1.44	1.19	1.15	1.10
60 × 10^−9^	0.90	1.29	1.19	1.11	1.00
50 × 10^−9^	0.88	1.24	1.16	1.12	1.07

## Data Availability

Not applicable.

## References

[1] Tomczak M. (1984). Defining marine pollution. Mar. Policy.

[2] Boelens R., Kershaw P.J., GESAMP (IMO/FAO/UNESCO-IOC/UNIDO/WMO/IAEA/UN/UNEP Joint Group of Experts on the Scientific Aspects of Marine Environmental Protection) (2015). Pollution in the Open Oceans 2009–2013—A Report by a GESAMP Task Team.

[3] (1991). GESAMP (Joint Group of Experts on the Scientific Aspects of Marine Environmental Protection): GESAMP Reports and Studies, No. 47.

[4] Gennaro M. (2004). Oil Pollution Liability and Control under International Maritime Law: Market Incentives as an Alternative to Government Regulation. Vanderbilt J. Transnatl. Law.

[5] Vikas M., Dwarakish G.S. (2015). International Conference on Water Resources, Coastal and Ocean Engineering (ICWRCOE 2015) Coastal Pollution: A Review. Aquat. Procedia.

[6] Geddes C.D., Lakowicz J.R. (2005). Rewiev in Fluorescence 2005.

[7] IMO The International Convention for the Prevention of Pollution from Ships (MARPOL), 1973 as Modified by the Protocol of 1978. http://www.imo.org/en/About/conventions/listofconventions/pages/international-convention-for-the-prevention-of-pollution-from-ships-(marpol).aspx.

[8] Fingas M. (2019). Marine Oil Spills 2018. J. Mar. Sci. Eng..

[9] Migliaccio M., Gambardella A., Tranfaglia M. (2007). SAR Polarimetry. To Observe Oil Spills. IEEE Trans. Geosci. Remote Sens..

[10] Hu C., Feng L., Holmes J., Swayze G.A., Leifer I., Melton C., García O., Macdonald I., Hess M., Muller-Karger F. (2018). Remote sensing estimation of surface oil volume during the 2010 Deepwater Horizon oil blowout in the Gulf of Mexico: Scaling up AVIRIS observations with MODIS measurements. J. Appl. Remote Sens..

[11] Robbe N., Zielinski O. (2004). Airborne remote sensing of oil spills-analysis and fusion of multi-spectral near-range data. J. Mar. Sci. Environ..

[12] Fingas M. (2012). The Basics of Oil Spill Cleanup.

[13] Zielinski O., Busch J.A., Cembella A.D., Daly K.L., Engelbrektsson J., Hannides A.K., Schmidt H. (2009). Detecting marine hazardous substances and organisms: Sensors for pollutants, toxins and pathogens. Ocean Sci..

[14] ESA (2017). Sentinel-1 Supports Detectionof Illegal Oil Spills. https://sentinel.esa.int/web/success-stories/-/sentinel-1-supports-detection-of-illegal-oil-spills.

[15] Brekke C., Solberg A.H. (2005). Oil spill detection by satellite remote sensing. Remote Sens. Environ..

[16] Zielinski O., Hengstermann T., Robbe N. (2006). Detection of oil spills by airborne sensors. Marine Surface Films.

[17] Otremba Z., Piskozub J. (2003). Modeling the remotely sensed optical contrast caused by oil suspended in the seawater column. Opt. Express.

[18] Haule K., Freda W., Darecki M., Toczek H. (2017). Possibilities of optical remote sensing of dispersed oil in coastal waters. Estuar. Coast. Shelf Sci..

[19] Haule K., Freda W. (2021). Remote Sensing of Dispersed Oil Pollution in the Ocean—The Role of Chlorophyll Concentration. Sensors.

[20] Otremba Z., Piskozub J. (2022). Monte Carlo Radiative Transfer Simulation to Analyse the Spectral Index for Remote Detection of Oil Dispersed in the Southern Baltic Sea Seawater Column: The Role of Water Surface State. Remote Sens..

[21] Baszanowska E., Otremba Z., Piskozub J. (2021). Modelling the Visibility of Baltic-Type Crude Oil Emulsion Dispersed in the Southern Baltic Sea. Remote Sens..

[22] Coble P.G. (1996). Characterization of marine and terrestrial DOM in seawater using excitation-emission matrix spectroscope. Mar. Chem..

[23] Coble P. (2013). Colored dissolved organic matter in seawater. Subsea Optics and Imaging.

[24] Drozdowska V., Wrobel I., Markuszewski P., Makuch P., Raczkowska A., Kowalczuk P. (2017). Study on organic matter fractions in the surface microlayer in the Baltic Sea by spectrophotometric and spectrofluorometric methods. Ocean Sci..

[25] Drozdowska V., Freda W., Baszanowska E., Rudź K., Darecki M., Heldt J., Toczek H. (2013). Spectral properties of natural and oil-polluted Baltic seawater—Results of measurements and modelling. Eur. Phys. J. Spec. Top..

[26] Kowalczuk P., Durako M.J., Young H., Kahn A.E., Cooper W.J., Gonsior M. (2009). Characterization of dissolved organic matter fluorescence in the South Atlantic Bight with use of PARAFAC model: Interannual variability. Mar. Chem..

[27] Miranda M.L., Mustaffa N.I.H., Robinson T.-B., Stolle C., Ribas-Ribas M., Wurl O., Zielinski O. (2018). Influence of solar radiation on biogeochemical parameters and fluorescent dissolved organic matter (FDOM) in the sea surface microlayer of the southern coastal North Sea. Elem. Sci. Anthr..

[28] Lopes R., Miranda M.L., Schuette H., Gassmann S., Zielinski O. (2020). Microfluidic approach for controlled ultraviolet treatment of colored and fluorescent dissolved organic matter. Spectrochim. Acta Part A Mol. Biomol. Spectrosc..

[29] McKee D., Röttgers R., Neukermans G., Calzado V.S., Trees C., Ampolo-Rella M., Neil C., Cunningham A. (2014). Impact of measurement uncertainties on determination of chlorophyll-specific absorption coefficient for marine phytoplankton. J. Geophys. Res. Oceans.

[30] Ostrowska M. (2012). Model dependences of the deactivation of phytoplankton pigment excitation energy on environmental conditions in the sea. Oceanology.

[31] Baszanowska E., Otremba Z. (2015). Modification of optical properties of seawater exposed to oil contaminants based on excitation-emission spectra. J. Eur. Opt. Soc. Rapid Publ..

[32] Baszanowska E., Otremba Z. (2017). Fluorometric index for sensing oil in the sea environment. Sensors.

[33] Baszanowska E., Otremba Z. (2019). Detecting the Presence of Different Types of Oil in Seawater Using a Fluorometric Index. Sensors.

[34] Baszanowska E., Otremba Z. (2020). Seawater fluorescence near oil occurrence. Sustainability.

[35] Kowalczuk P., Stedmon C.A., Markager M. (2006). Modeling absorption by CDOM in the Baltic Sea from season, salinity and chlorophyll. Mar. Chem..

[36] Jerlov N.G. (1976). Marine Optics.

[37] Del Vecchio R., Blough N.V., Ghetti F., Checcucci G., Bornman J.F. (2005). Influence of ultraviolet radiation on the chromophoric dissolved organic matter in natural waters. Environmental UV Radiation: Impact on Ecosystems and Human Health and Predictive Models, Proceedings of the NATO Advanced Study Institute, Pisa, Italy, June 2001.

[38] Hargreaves B.R., Helbling E.W., Zagarese H. (2003). Water column optics and penetration of UVR. UV Effects in Aquatic Organisms and Ecosystems.

[39] Ecohydrodynamic Forecast for the Baltic Sea. http://model.ocean.univ.gda.pl/php/frame.php?area=ZatokaGdanska.

[40] Drozdowska V., Józefowicz M. (2015). Spectrophotometric studies of marine surfactants in the southern Baltic Sea. Oceanologia.

[41] Kowalczuk P., Darecki M., Zabłocka M., Górecka I. (2010). Validation of empirical and semi-analytical remote sensing algorithms for estimating absorption by Coloured Dissolved Organic Matter in the Baltic Sea from SeaWiFS and MODIS. Oceanologia.

